# Antibiotic prescribing practices, perceived constraints, and views on antimicrobial resistance among general and orthopedic surgeons in central India

**DOI:** 10.1038/s41598-025-11173-w

**Published:** 2025-07-11

**Authors:** Kristina Skender, Anna Machowska, Shweta Khare, Vivek Singh, Cecilia Stålsby Lundborg, Megha Sharma

**Affiliations:** 1 Department of Global Public Health, Health Systems and Policy, Karolinska Institutet, Stockholm, 17177 Sweden; 2 Department of International Centre for Health Research, Ruxmaniben Deepchand Gardi Medical College, Ujjain, 456006 India; 3 Department of Orthopedics, Ruxmaniben Deepchand Gardi Medical College, Ujjain, 456006 India; 4 Department of Pharmacology, Ruxmaniben Deepchand Gardi Medical College, Ujjain, 456006 India

**Keywords:** Qualitative, Antibiotic prescribing, Antimicrobial resistance, Perceptions, Surgery, Orthopedic, India, Public health, Bacterial infection, Orthopaedics, Health policy

## Abstract

India has one of the world’s highest burdens of antimicrobial resistance (AMR). Broad-spectrum antibiotics are routinely and empirically initiated in surgery and orthopedic departments, contributing to the AMR. This study aimed to explore the capabilities, opportunities and motivation that influence antibiotic prescribing practices of general and orthopedic surgeons in Central India. This qualitative study was guided by the Capability, Opportunity, Motivation-Behavior model (COM-B) and the Theoretical Domains Framework (TDF). Semi-structured interviews were conducted until redundancy was achieved, including 15 general and orthopedic surgeons in three private-sector hospitals in Ujjain district. Questions were formulated to explore how decisions about antibiotic prescribing are made, and surgeons´ perceptions of antibiotic use, AMR and potential solutions. Manifest and latent content analysis was used. Three main themes were revealed: (1) Antibiotic prescribing decision is a multifactorial process influenced by environmental and sociocultural factors; (2) Infection prevention and control (IPC), diagnostics and treatment need strengthening; (3) AMR is a social problem that requires a collective effort. Participants emphasized the critical role of environmental (e.g., dusty rural setting) and sociocultural factors (e.g., patients’ socioeconomic status) in influencing antibiotic prescribing decisions. Prescribing practices were mostly empirical and varied between participants. Junior practitioners expressed a strong desire for regulation, guidelines, feedback and improvement, whereas senior practitioners were more confident in their prescribing. Doctors expressed frustration and hopelessness when treating resistant infections and recalled the significant contribution of the COVID-19 pandemic to inappropriate antibiotic use. The AMR problem in India was perceived as a broader social issue requiring collective action. Proposed solutions included strengthening IPC and diagnostic practices, developing contextualized antibiotic prescribing guidelines, stricter regulations on antibiotic use, and public education. Antibiotic prescribing decisions are multifactorial and context-specific, strongly impacted by prescribers’ experiences and environmental and sociocultural factors. Our findings could inform future interventions to improve antibiotic use in Central India and beyond.

**Supplementary Information:**

The online version contains supplementary material available at 10.1038/s41598-025-11173-w.

## Introduction

Antimicrobial resistance (AMR) is a silent pandemic that particularly affects low- and middle-income countries (LMICs) in South Asia^1,2^, with India having one of the highest AMR burdens in the world^3,4^. The high burden of AMR in India is largely driven by overconsumption of antibiotics, which is influenced by high infectious disease burden, inadequate infection prevention and control (IPC) measures, overburdened health system, over-the-counter (OTC) sales of antibiotics, limited laboratory resources, the absence of guidelines and surveillance systems for antibiotic use in healthcare facilities, a lack of awareness among healthcare providers and the public, and incentives offered by pharmaceutical companies for antibiotic prescriptions^3–6^.

Globally, antibiotic prescribing practices often do not align with the evidence-based guidelines^7,8^. It is estimated that over one-third of antibiotic prescriptions in hospitals worldwide are inappropriate^9^. One of the global goals is to reduce antibiotic use in healthcare. This often requires changing behaviors, with interventions designed to influence the knowledge and attitudes of prescribers^10^. A major challenge for clinicians is the need to make rapid decisions regarding empirical treatment based on signs and symptoms of infection, aiming to reduce morbidity and mortality for individual patients. However, this approach also contributes to the long-term problem of AMR, as it involves administering treatment without knowing the causative pathogen or having a definitive diagnosis^9,11^. Therefore, clinicians must balance patient and public health benefits when prescribing antibiotics, while also navigating various influencing factors.

In surgery and orthopedics, broad-spectrum antibiotics are routinely used and largely empirically initiated, to prevent or limit the infection^12,13^. Perioperative antibiotic prophylaxis is a main reason for antibiotic use in hospitals, often showing high rates of inappropriate prescribing, such as extended duration of antibiotic prophylaxis^14^. Some commonly reported reasons for inappropriate antibiotic prescribing in surgery include diagnostic uncertainty, prescriber’s lack of training or experience, complex comorbidities, unfamiliarity with local resistance patterns, and mistakes in interpreting microbiological results^15^. Furthermore, research from Australia has identified several factors contributing to surgeons’ non-compliance with antibiotic prescribing guidelines, such as concern about patient outcomes, the prioritization of technical surgical skills over antibiotic management skills, mistrust in the guidelines, the acceptance of improvisation in antibiotic prescribing, etc^14,16^. However, most of these findings come from high-income countries, and there is limited understanding of the factors driving antibiotic prescribing behavior in surgical departments in LMICs^17^. Research has shown a strong association between inappropriate antibiotic prescribing and financial as well as sociocultural contexts^9^.

The majority of India’s health infrastructure, including 93% of all hospitals, belongs to the private sector^18,19^, which is often slower than the public sector in implementing national guidelines and health policies^20^. Previous quantitative studies in surgery and orthopedic departments of private hospitals in Central India revealed suboptimal prescribing practices; however, the underlying reasons remain unclear^21–23^. Therefore, the objective of this study was to explore the capabilities, opportunities and motivation of general and orthopedic surgeons that influence their antibiotic prescribing behavior in private-sector hospitals in Central India.

## Methods

### Study design

This qualitative study employed semi-structured interviews and was based on the integrated Capability, Opportunity, Motivation-Behavior (COM-B) model and the Theoretical Domains Framework (TDF), widely used in behavior change research of healthcare workers^24^. COM-B identifies three main components that interact to generate behavior: capability, opportunity and motivation. COM-B is linked with the 14 domains of the TDF used to describe behavior: knowledge; skills; social/professional role and identity; beliefs about capabilities; optimism; beliefs about consequences; reinforcement; intentions; goals; memory, attention, and decision processes; environmental context and resources; social influences; emotion; behavioral regulation^24^ (Table S1).

### Study setting

The study was conducted in Ujjain district, Madhya Pradesh. Madhya Pradesh is the second-largest state in India by area and the fifth-largest by population, with 72 million residents, approximately 72% of whom live in rural areas^25^. The 2023 India’s National Multidimensional Poverty Index ranked Madhya Pradesh as the fifth-poorest of 28 states in India, with 21% of its population living in poverty^26^ and 64% literacy rate^25^. Compared to the Indian average, Madhya Pradesh has poorer health indicators, including higher maternal mortality (173 per 100,000 live births), infant mortality (46 per 1,000 live births), and under-five mortality (56 per 1,000 live births)^25^. Ujjain district has 1.9 million inhabitants, out of which 61% live in rural areas and are primarily working in agriculture. The population literacy rate is 72%, which is below the national average. In the Ujjain district, 77% of villages lack public healthcare facilities^27^.

Interviews were conducted in the orthopedic and surgery departments of three private-sector hospitals in the Ujjain district: CR Gardi Hospital (CRGH), Ujjain Charitable Trust Hospital (UCTH) and Avanti Hospital (AH). CRGH is a teaching hospital affiliated with Ruxmaniben Deepchand Gardi Medical College, located on the outskirts of Ujjain city, with a capacity of 650 beds. UCTH and AH are situated in the city center, with capacities of 200^20,28^ and 150 beds, respectively^29^. All hospitals are part of the same not-for-profit charitable trust. Each hospital provides laboratory services, which were subsidized at the CRGH, whereas UCTH and AH charged nominal fees for laboratory diagnostics^20,28^.

### Participant selection and data collection

For this study, 15 doctors were selected, including 8 general and 7 orthopedic surgeons from the three study hospitals. Suitable participants were identified by homogenous purposeful sampling method. Participants were contacted both by telephone and face-to-face to organize the interview schedule. None of the contacted participants refused to participate or dropped out. Participant selection was planned to obtain a variation of age, education and experience level. Work experience was recorded from the time when participants began practicing medicine, regardless of whether they immediately pursued a specialization in orthopedics or surgery. Senior consultants and professors were classified as seniors, whereas residents in orthopedics and surgery and junior consultants were referred to as juniors. As most of the general and orthopedic surgeons were male, the equitable variation in sex could not be achieved (Table 1.). Quiet space in each hospital was identified to conduct most interviews; however, some interviews had to be conducted in the clinicians’ outpatient offices and were occasionally interrupted by consultants and nurses.

**Table 1:** Participants’ demographic details.

Participant number	Sex	Age	Department	Seniority level	Work experience
1	Male	29	Orthopedics	Junior	8 months
2	Male	27	Orthopedics	Junior	2 years
3	Male	26	Orthopedics	Junior	2.5 years
4	Male	28	Orthopedics	Junior	2.5 years
5	Male	40	Orthopedics	Senior	15 years
6	Male	> 50	Orthopedics	Senior	> 25 years
7	Male	33	Orthopedics	Senior	3.5 years
8	Male	52	Surgery	Senior	24 years
9	Female	> 50	Surgery	Senior	> 25 years
10	Male	35	Surgery	Junior	7 years
11	Male	46	Surgery	Senior	18 years
12	Female	27	Surgery	Junior	2 years
13	Male	28	Surgery	Junior	3 years
14	Male	> 50	Surgery	Senior	> 25 years
15	Female	26	Surgery	Junior	1 year

Semi-structured interviews were conducted in English in March 2023 by K.S. This interview method allowed prescribers to speak openly about their antibiotic prescribing practices and to include all the important aspects of antibiotic prescribing behavior in the given time. Questions were resourced from a topic guide for interviews about antibiotic use in hospitals, developed by Tarrant et al.^9^, and adapted to the study’s aim and context by. K.S. and M.S. Furthermore, questions were formulated to explore and address crucial moments in the clinical decision-making process on antibiotic prescribing^30^. The interview guide contained open-ended questions and prompts (Table S2.), that were pilot tested with one orthopedic surgeon before final use. Field notes were taken during and immediately after the interviews. During the course of the interviews, some new topics emerged, which appeared to be relevant to the participants and were thus added to the interview guide. The following areas were covered in the interview guide: how decisions about the need for antibiotic prescribing are made; decisions regarding the type of antibiotic and the duration of treatment; the practice of sending samples to the laboratory; perceptions of the quality of medical treatment and antibiotic prescribing practices; perceptions of the AMR problem and potential solutions; and antibiotic prescribing practices during the COVID-19 pandemic. Interviews lasted from 16 to 47 min (mean duration 31 min). Data collection continued until redundancy was achieved. All interviews were audio recorded and transcribed verbatim and cross-checked for the correctness of the text.

## Analysis

Interviews were coded by K.S. and cross-checked by S.K., both trained in qualitative methods. Discrepancies in coding were resolved by discussion with a senior researcher, M.S. Coding process and analysis were based on manifest and latent content analysis, as described by Graneheim et al.^31,32^. A variation of different types of codes (in-vivo, emotions, values, actions, behaviors) was created by arising directly from data. Field notes and memos created during data collection and analysis helped in the interpretation and organization of the meaning units into codes, (sub)categories and (sub)themes. Interview topics were broadly identified in advance, but as new topics emerged from the data during analysis, final themes were identified through an iterative process of moving between codes, (sub)categories and (sub)themes. NVivo software was used during the process of analysis. Results of the study were presented to the participants for member check and feedback. The COREQ qualitative research reporting checklist (Suppl. Material 2) was used to achieve thorough reporting.

### Ethical considerations

Information sheet and informed consent form were created based on the World Health Organization (WHO) template for informed consent for qualitative studies^33^. Interviewees were briefed about the interviewer’s role and purpose of the study before the interview and gave written and oral consent to participate and record the interview. Participants were informed about their right to withdraw from the interview at any time and agreed to provide their emails so that the interviewer could contact them if any questions arise. All information was treated as confidential. Sound files and transcripts were stored in the password-protected computer in India with personal identifiers removed. Data was analyzed pseudo-anonymously, and participants were coded by their department (S for surgery, O for orthopedics) and a number.

## Results

**Fig. 1:**
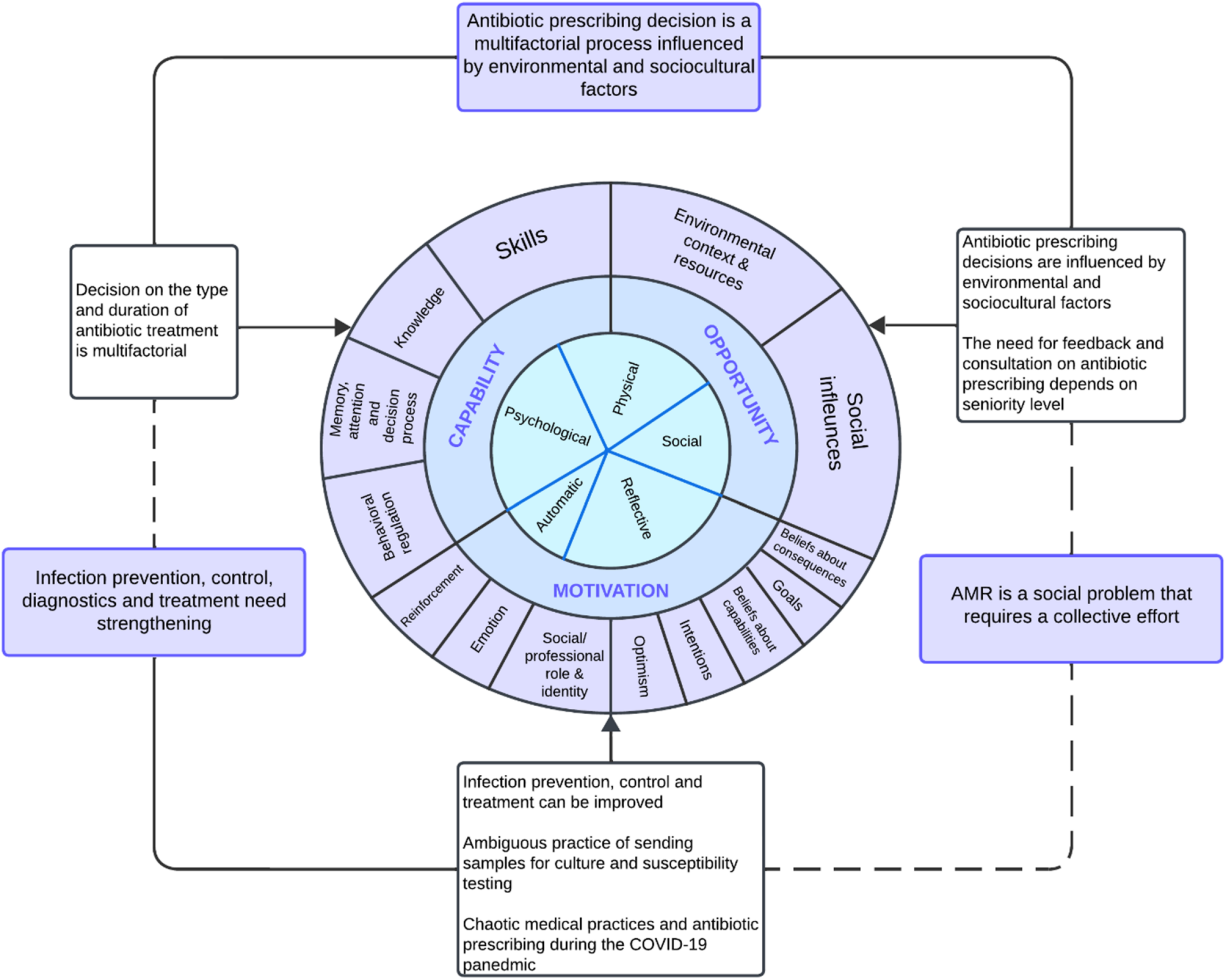
Main themes and subthemes mapped against COM-B-TDF framework.

Data analysis uncovered three main themes: (1) Antibiotic prescribing decision is a multifactorial process influenced by environmental and sociocultural factors; (2) IPC, diagnostics and treatment need strengthening; (3) AMR is a social problem that requires a collective effort. The analysis process that led to the creation of (sub)themes from (sub)categories, codes and meaning units is presented in the coding tree (Table 2). Themes and subthemes were mapped against the COM-B-TDF framework (Fig. 1).

**Table 2:** The coding tree.

Theme	Subtheme	Category	Subcategory	Code	Condensed meaning unit	Meaning unit
Antibiotic prescribing decision is a multifactorial process influenced by environmental and sociocultural factors	Antibiotic prescribing decisions are influenced by environmental and sociocultural factors	Patients’ education and behavioral factors that influence treatment	Patients’ pressure and beliefs around antibiotics	Patients believe that treatment without antibiotics is incomplete	Patients have been brainwashed to believe that treatment without antibiotics is incomplete	*“Yes*,* patients have been brainwashed…like if you are not giving them antibiotics*,* if you are not IV or oral*,* then your treatment is incomplete.” (O-05)*
	Decision on the type and duration of antibiotic treatment is multifactorial	Choice between broad-spectrum and narrow-spectrum antibiotic	Benefits of broad-spectrum antibiotics	Effective	Experience of good results	*“I have seen some good results*,* so I prescribe them.” (O-07)*
	The need for feedback and consultation on antibiotic prescribing depends on seniority level	Need to ask for advice on antibiotic prescribing	Cases in which there is a need for advice	If infection is not controlled even after changing the antibiotic based on the culture and susceptibility report	Seeking advice in cases of uncontrolled infection and when local wounds are getting more infected after the blood culture and susceptibility report	*“For the cases*,* usually where the uncontrolled infection is there*,* where the local wounds are getting infected*,* for the cases where we have a growth or we can’t find any other mitosis except for the local factors. So*,* for those cases when we have a proper blood culture report*,* we go for a discussion for that thing.” (S-05)*
IPC, diagnostics and treatment need strengthening	Infection prevention, control and treatment can be improved	Perception of IPC practices	Need for better IPC measures in operating theatre	Operating theatre environment is not well regulated	Less control over environmental factors in the operating theatre which leads to hospital-acquired infections	*"Sometimes hospital-acquired infection post-operative. Linen, proper environment, temperature, humidity. There are less control over all these things." (O-06)*
	Ambiguous practice of sending samples for culture and susceptibility testing	Importance and objectives of sending samples for culture and susceptibility testing	Targeted treatment	Antibiotics should not be used randomly	Medicines and antibiotics should not be used randomly	*“So*,* I don’t want to use any medicine randomly. Any antibiotic randomly should not be used”. (S-02)*
	Chaotic medical practices and antibiotic prescribing during the COVID-19 pandemic	Misuse of antibiotics and steroids in COVID-19 times	Government was delivering Coronavirus kits to homes	People were isolating and taking medications at home	People were convinced that they were infected, so they were isolating and taking medications at home	*“Even when you are not infected*,* you know that you are infected. So*,* you are taking medication*,* you are home isolated. You are taking medication at home."(S-06)*
AMR is a social problem that requires a collective effort	AMR requires a collective effort	Ways of reducing AMR	Regular education, collaboration and meetings about antibiotic use and development of resistance	Senior consultants should be regularly updated on new protocols and guidelines	When there is a new guideline, prescribers should be aware of it	*“Most of the times*,* whenever we get a new guideline and then…the persons who are having authority to prescribe these antibiotics should be aware of these guidelines.” (O-04)*

### Theme 1- antibiotic prescribing decision is a multifactorial process influenced by environmental and sociocultural factors

General and orthopedic surgeons indicated that their antibiotic prescribing decisions were largely guided by medical knowledge and clinical assessment of factors such as the type and severity of injury, type of surgery and signs of infection. Interviewees felt they made good decisions about antibiotic prescribing based on patient follow-up, when it showed improvement in the patient’s condition. They also emphasized the critical role of antibiotics in surgical care, expressing views like, *“antibiotics are most important in orthopedic surgeries” (O-02)* and *“surgical management is all about injectable antibiotics” (S-12).* Furthermore, special considerations that affect their clinical decision-making on antibiotic prescribing were revealed, including the desire to achieve positive patient outcomes despite diagnostic uncertainty and the specific challenges of treating orthopedic infections. Orthopedic surgeons noted that treating complications in orthopedics is often a long and painful process with uncertain outcomes.

#### Antibiotic prescribing decisions are influenced by environmental and sociocultural factors

Throughout the interviews, context-specific environmental and sociocultural factors influencing antibiotic prescribing emerged as a major topic. It was often mentioned that dry and dusty rural environment *“pushes the use of antibiotics” (S-08).* Participants felt that many more factors influence clinical decisions on antibiotic prescribing in India compared to high-income countries, such as a high patient load and excessive workload for doctors, the quality of medical education and available facilities, and the influence of pharmaceutical companies on doctors’ and patients’ behavior. Furthermore, interviewees highlighted patients’ socioeconomic status as a critical factor affecting antibiotic prescribing and patient recovery in India. It was frequently mentioned that patients in the area were poor; therefore, the affordability of antibiotics was a major factor in treatment decisions, along with the costs of diagnostics and surgeries. Furthermore, it was often noted that a patient’s compliance and trust in the doctor depended on the affordability of the prescribed antibiotics. Consequently, doctors often needed to modify their prescriptions, sometimes opting for simpler, less expensive antibiotics even when patients needed “higher” broad-spectrum antibiotics, which can be very costly.

*“If we have to switch to the higher antibiotics like the piperacillin-tazobactam*,* the combination ones*,* two-three antibiotics at a time…So*,* these are the very costly ones*,* most patients cannot afford. Instead of that*,* they choose to quit the hospital…You just give whatever you can. So*,* we have to switch to a lower spectrum antibiotic. Even if it’s not working. Because sometimes you know this antibiotic [is] resistant*,* even then we are prescribing it because we don’t have any other option. We cannot go to get colistin for every patient. The last option left for all resistant patients is colistin. But one injection costs around 1000 bucks. And their monthly income is 1000 rupees. So*,* they cannot afford it. So*,* we have to give something*,* even if it is resistant. So at least for moral grounds*,* we are treating the patient. Otherwise*,* it is just that the patient is left on their own.” (S-13)*.

Further significant factors that influence clinical decision-making on antibiotic use were patient education and behavioral factors: illiteracy, poor hygiene practices, late presentation, loss to follow-up, low treatment compliance, a desire to get better instantly, and patients’ pressure and beliefs about antibiotics. For example, patients believed that the treatment without antibiotics was incomplete and consequently, doctors were perceived better if they prescribed more antibiotics and steroids. In general, *“the more antibiotics*,* the better” (O-05)* was a wide-spread patient belief.

*“…and in private practice*,* if patient is not responding very well*,* means no response to our treatment*,* patient will switch over to other fellow*,* other doctor… So*,* they want miracles. Within a week they want to be cured.” (S-09)*.

#### Decision on the type and duration of antibiotic treatment is multifactorial

The choice of antibiotic was found to be influenced by multiple factors, including the patient’s condition and history, the antibiotic’s pharmacokinetic and pharmacodynamic properties, the prescriber’s experience, and personal preference. Prescriber experience was particularly emphasized, with many interviewees expressing that they relied on their knowledge of the most common organisms causing infections to guide their antibiotic selection. Similarly, personal preferences in prescribing were highlighted, as participants frequently mentioned that different doctors had different prescribing practices, such as preference of starting with oral antibiotics, using only broad-spectrum antibiotics, or using antibiotics only for operative cases, etc.

*“The only thing is*,* few antibiotics have been inoculated in our culture*,* in our prescription culture*,* because of our practices*,* which we have seen*,* like which our seniors have seen through their experiences*,* that this antibiotic is working well in such scenario.” (S-12)*.

Surgeons generally favored broad-spectrum antibiotics due to their effectiveness and ability to cover a wide range of bacteria. The treatment was most frequently initiated with broad-spectrum antibiotics to control and prevent the spread of infection because clinicians *“cannot individualize antibiotic therapy from day one” (O-05).* The use of broad-spectrum antibiotics was considered standard practice in the surgical field, with many interviewees having positive experiences with broad-spectrum antibiotics in their setup.

*“Otherwise*,* from my experience of last 23 years*,* I have operated so many cases of perforation*,* obstruction*,* but this is my experience that I can say proudly that I have saved most of the patients. And definitely*,* there is a role of this broad-spectrum antibiotics.” (S-08)*.

The most emphasized benefit of narrow-spectrum antibiotics was their ability to provide targeted treatment. However, participants rarely started the treatment with narrow-spectrum antibiotics, except in cases of minor injuries or surgeries, due to uncertainty about their effectiveness. When asked about how often they switched from broad-spectrum to narrow-spectrum antibiotics, responses varied widely. Some reported doing so in most cases, while others stated it happened only in very few cases, as they typically did not switch to narrow-spectrum if broad-spectrum antibiotic was effective. Reasons for switching from broad-spectrum to narrow-spectrum antibiotics were similarly inconsistent. Some interviewees opted to switch when the patient’s condition was improving, while others did so when the condition was not improving, guided by culture and susceptibility reports.

Surgeons reported a wide range of antibiotics they commonly prescribed, with amoxiclav, aminoglycosides and third-generation cephalosporins being the preferred choices. They generally started treatment with third-generation cephalosporins, with the addition of amikacin and metronidazole for contaminated wounds or major injuries.

In terms of decisions on the duration of antibiotic treatment, interviewees considered the following factors: the type and severity of the injury, IPC measures, and the type of surgery. The duration of perioperative surgical prophylaxis was not standardized and ranged from one to three doses. Many surgeons acknowledged that determining the duration of antibiotic treatment was problematic and not defined. Intravenous broad-spectrum antibiotics were generally given for two to five days, with extended durations in major surgeries and severe cases, particularly in certain orthopedic conditions (e.g., osteomyelitis and septic arthritis). Oral antibiotics were commonly prescribed for two to three days during hospitalization and five to seven days after discharge. Some participants revealed that they prescribed antibiotics for longer durations due to fear of postoperative infections or patients not returning for follow-up.

*“Sometimes for the patient*,* for our own mental peace also*,* we step up the antibiotic so that we are avoiding an unforeseen infection*,* which is not justifiable*,* because what if that infection never occurred? So*,* I think we need to have a protocol for this thing also…So if someone tells us or some kind of proof comes that our antibiotics*,* even oral antibiotics*,* can work or if we step down then it won’t be an issue. Then I think that will help us to at least decrease our use in antibiotics for the duration. If someone tells us that even a 3-day duration is enough and we have a proof for that*,* then we can try that. We need not go for a 5-day duration.” (S-12)*.

#### The need for feedback and consultation on antibiotic prescribing depends on seniority level

When asked whether they followed any guidelines for antibiotic prescribing and which ones, some participants stated that they adhered to international and national guidelines, as well as a “general standard protocol” or “departmental and hospital guidelines.” However, most participants indicated that there were no formal guidelines for antibiotic prescribing in India, nor written departmental or hospital guidelines, stating that *“there cannot be universal guidelines for every patient in every part of the world” (O-05).*

There was considerable variation in responses regarding feedback on antibiotic prescribing. Some interviewees reported not receiving regular feedback, while others indicated they received feedback primarily from seniors, colleagues, and patients. The majority of participants viewed regular feedback as valuable for optimizing antibiotic treatment and expressed openness to regular prescription audits.

*“It [feedback]would be helpful for us because now*,* since we are a team*,* we have a different mode of treatment*,* different types of patients. So*,* we can generalize this and then we can get into practice*,* whichever is most favorable among them [antibiotics]. That would be good for us.” (S-03)*.

The responses regarding the need to seek advice on antibiotic prescribing were similarly varied. While some senior participants felt confident in their prescribing decisions and seldom, if ever, sought advice, others indicated they consulted microbiology, pharmacology, internal medicine, or pathology departments for complex infectious cases. Most junior residents, however, reported regularly seeking guidance from senior consultants and professors.

*“Like our consultants*,* usually when we are on a round…*,* they always explain us why we need to change the antibiotic or what is the protocol*,* why you should change it or which to start and which to stop and for how many days*,* which is more important.” (S-05)*.

### Theme 2- infection prevention and control, diagnostics and treatment need strengthening

#### Infection prevention, control and treatment can be improved

Some surgeons pointed out issues with the operating theater conditions and the preoperative preparation of both surgeons and patients. It was also noted that overall cleanliness of the hospitals could be greatly improved, as patients were considered more likely to acquire infections after leaving the operating theater than within it. When reflecting on surgical site infections (SSIs), many participants noted that infection rates were low in both orthopedic and surgical departments. However, when SSIs did occur, they highlighted the severity of the consequences, describing them as a *“disaster”* and a *“failed surgery” (O-06).*

Most surgeons saw their antibiotic prescribing practices as good and justified, mentioning that they did not overuse antibiotics and were very restricted on the use of “higher” antibiotics. Several interviewees stated that there was no external pressure or restrictions on the use of antibiotics. Senior surgeons reflected on how antibiotic prescribing has improved over time through education from conferences and symposiums and acknowledged that “*20 years ago*,* we were using much more antibiotics than today” (S-08).* Junior surgeons appeared to be more critical of departmental antibiotic prescribing practices and saw room for improvement. It was often mentioned how their prescribing is based on theoretical knowledge from medical books, as well as on what senior doctors had taught them from their experience.

*“In our setup*,* I think the protocols need to be a bit strict…For the broad-spectrum antibiotics*,* there should be a teaching at least*,* and also an explanation*,* like not at the level of junior residents. It should begin at the level of consultant because the one who teaches is the one who should be learned most…Because antibiotics is a thing*,* the protocol changes. So*,* though they know everything*,* but still they need to be updated on this. There should be a program or something that keeps them updated every 3–6 months so that they can keep us updated on these things. Because if you teach a junior resident*,* they can’t impose things unless their superior is ready for it. So*,* the things should begin from the top level…The channel should be that way*,* it should not go the other way. Because residents can never have a say in anything unless the consultants are ready for it. Even justified use of antibiotics should be understood from their levels.” (S-12)*.

#### Ambiguous practice of sending samples for culture and susceptibility testing

Most surgeons stated that their standard practice was to send a sample for culture and susceptibility testing before empirically starting broad-spectrum antibiotics, with treatment later adjusted based on the test results. However, there was significant ambiguity regarding whether samples are consistently sent for culture and susceptibility testing, and if so, the exact timing of when the samples are sent remained unclear.

Participants noted they used various clinical criteria to decide whether to send samples for culture and susceptibility testing, though these criteria differed among them. As a result, their responses regarding the frequency of sending different types of samples varied. For instance, the reported frequency of sending blood cultures ranged from “in very few cases” to “in all infected cases.” Similarly, the frequency of sending pus cultures ranged from “rarely” to “very often.”

Most surgeons believed that sending samples for culture and susceptibility testing was very important for ensuring evidence-based, targeted, and appropriate antibiotic use, as well as for investigating local resistance patterns.

*“You need to know what are the bugs around and if you know what are the bugs around then you can treat them properly. Otherwise*,* it is all shot in the dark.” (S-14)*.

Although many participants were, in general, satisfied with laboratory services in hospitals, they identified several problems related to the practice of sending samples for culture and susceptibility testing. Some interviewees complained about the long waiting time for results, which was generally two to three days but could be delayed up to five days with an increased sample load. In addition, some participants indicated the occasional lack of resources in the departments, e.g. sample tubes and gloves. Several participants revealed that they sometimes could not trust culture and susceptibility reports due to inappropriate handling of samples, such as contamination during collection or transit, or misplacement of samples due to errors in labeling and identification.

Various suggestions were proposed to improve the process of sending samples for culture and susceptibility testing. The need for the development and implementation of a standardized sample collection protocol was emphasized, along with regular training and supervision of staff on proper procedures for collecting, transporting, and storing samples. Similarly, there was a call for clear guidelines on the types of cases and the frequency for mandatory sending of samples, both before initiating and during antibiotic treatment, to ensure appropriate use. Some interviewees suggested expanding the range of antibiotics tested for susceptibility, as it is *“no use for a clinician to get the report which is resistant to all antibiotics” (O-05).* Additionally, improvements in the notification system from the laboratory to clinicians regarding sample reception and culture results were suggested. It was highlighted that every unit and department should have sufficient stocks of necessary materials, such as sample tubes. Ideally, some surgeons proposed that each department that frequently requires laboratory services should have its own laboratory, and that faster, alternative diagnostic methods should be developed.

#### Chaotic medical practices and antibiotic prescribing during the COVID-19 pandemic

Many participants evoked the topic of COVID-19 and its impact on their medical practices, particularly in relation to antibiotic prescribing. One senior surgeon noted that during the pandemic, he encountered unusually severe infections where the causative pathogens were unidentified, leading to the highest mortality rates he had ever seen in his practice. Participants recalled the uncertainty they were facing when deciding about treatment protocols, which were frequently changed by the WHO and national health authorities. It was often stressed how antibiotics and steroids were largely misused during pandemic, as broad-spectrum antibiotics were given to every patient, and how people were self-medicating and randomly buying medicines OTC. Additionally, the government distributed “Coronavirus treatment kits” to COVID-19 suspected and confirmed cases. These kits contained antibiotics (azithromycin, doxycycline) and steroids, which people were taking while isolating at home. The overall situation was described as *“bizarre-everything was going on” (S-13).*

*“… and organisms developing a resistance because of mismanagement at the level of antibiotic because which has occurred during COVID. During COVID*,* no one knew what would work. So*,* everyone has tried every antibiotic on almost many patients and that has helped the viruses and the bacteria to outgrow such antibiotics.” (S-12)*.

### Theme 3- AMR is a social problem that requires a collective effort

Surgeons shared their observations regarding the development of AMR in their practice, noting that bacteria have become resistant not only to “common basic antibiotics”, but also to broad-spectrum antibiotics, such as third-generation cephalosporins and carbapenems. Most participants said that they were now more aware of the AMR and expressed concern, stating that there is *“no assurance anymore that we will be on the safe side when giving a broad-spectrum antibiotic” (O-05).* The development of AMR was reflected in the need to change the prescribing protocol every 10 to 15 years. When asked about how often they encounter AMR, most participants indicated that they rarely experienced resistance to all antibiotics, though they acknowledged that the trend of resistance to all antibiotics is increasing. Nonetheless, many reported frequently encountering patients with resistance to some antibiotics, particularly “common antibiotics”.

*“So very rarely do we find a bacteria or culture which is sensitive to a common antibiotic. Nowadays we get patient which are sensitive to…colistin*,* tigecycline.” (O-05)*.

Perceived causes of AMR development were misuse of antibiotics, namely overprescription, multiple treatments, patient non-adherence to prescribed regimens, extended duration of antibiotic therapy not based on evidence, and *“easily stepping up*,* but afraid of stepping down” (S-12)*, as described below.

*“Antibiotic prescription*,* somewhat I think*,* needs an improvement in our practices because though we have some established dictums in our mind that these kind of antibiotics [work]*,* we don’t step down easily. We step up our antibiotic usage easily*,* we don’t step down.” (S-12)*.

An important contributing factor to the development of AMR, as identified by many interviewees, was that patients had already been treated with antibiotics somewhere else before coming to the hospital. Some participants placed the main fault for AMR development at primary and secondary healthcare facilities and described their practices as the random use of antibiotics by untrained personnel, indicating frequent treatment of the common cold with antibiotics. Similarly, fully private clinics were seen as contributing more to AMR than their hospitals due to a lack of supervision and specialized doctors.

*“You can buy antibiotics over-the-counter in India. It is not a big thing. It’s very much irrational use of antibiotics in the peripheral zones. Patients first take the treatment in the periphery. They are getting antibiotics from the quacks and everything. They are just prescribing everything*,* steroids*,* antibiotics*,* antacids and everything. And then when they do not improve*,* they come to us. So*,* they have already taken the antibiotics*,* and we don’t know what they have taken. So*,* we have to start on our judgment.” (S-13)*.

While discussing the AMR problem, many participants compared their setting in India to high-income countries and mentioned some of the contributing factors to the high AMR burden in India, like unrestricted availability of OTC antibiotics, high production and use of antibiotics, and AMR being more of a social problem, rather than being solely caused by doctors’ treatment.

*“But in countries who are developing or not developed*,* a country like us*,* India*,* the most important thing we should start is health promotion*,* first thing. Healthy practices. Until and unless we will teach the people*,* it is not possible to achieve this goal. We are doing many things from our part. We are doing each and everything. We are taking cultures*,* we are prescribing antibiotics*,* targeted antibiotics*,* broad spectrum. What not we are doing. But again*,* we have treated the patient*,* he has survived*,* we have made efforts*,* and patient has survived and now again what he will do? He will go in the same scenario. So*,* what will happen? He is not knowing the exact value of cleanliness and healthy practices. This is the most important thing which should be done worldwide. It is basically a social problem because a trained person will not do deliberately all these things…So on mass scale*,* it is very necessary to promote such practices in the remotest area of the world.” (S-08)*.

Surgeons reflected on AMR’s influence on patients, by saying that although most patients recover, it profoundly affects every aspect of patients’ lives. The patient’s physical state is affected by the deterioration of the condition, complications, prolonged hospitalization and treatment. In addition, it was also mentioned how AMR negatively affects patient’s mental health and social and family life. Furthermore, the big economic impact of AMR on patients was repeatedly highlighted, as “higher” antibiotics, like colistin and tigecycline, are very costly.

In terms of how AMR influences doctors, interviewees mentioned that the occurrence of AMR does not reflect well on them as healthcare professionals. Surgeons expressed a sense of responsibility for their patients, and feelings of stress, frustration, *“tied hands” (O-05)*, hopelessness and failure when dealing with AMR, which was described as *“nightmare for patients and us”(O-04)*.

*“What can we do? Our hands are tied. We don’t give antibiotics for fun! I mean*,* we have to give some antibiotics…in case of an open fracture*,* to limit the spread of infection. If it is not infected*,* to limit the infection…We have to give some antibiotics. But what antibiotics? Our hands are tied. We are just part of the community.” (O-05)*.

*“We have investigated*,* culture is resistant to all the antibiotics*,* then what should we do? We cannot sit. We cannot sit and wait and see patient to die. So*,* there is*,* because there is no guidelines at present*,* what should be done to such cases? Most of the time we start some higher broad-spectrum antibiotics.” (S-08)*.

Although most surgeons were uninformed about the extent of the AMR burden in their departments and hospitals, they often discussed the treatment of resistant cases in departmental meetings. They emphasized how resistant infections were very challenging to treat and required change of case management plan, sending repeated cultures, prescription of “higher” broad-spectrum antibiotics (e.g. carbapenems, colistin, tigecycline etc.), non-pharmacological treatment (e.g., repeated cleaning and dressings of the wound, drainage of the abscess, debridement of the bone and joint, etc.) and in some cases referral of the patient. Some participants revealed that when “higher” antibiotics were not working, they experienced “basic” older antibiotics to be effective, like sulfamethoxazole-trimethoprim.

In discussing ways to reduce AMR, a *“collective effort from everybody” (S-13)* approach was suggested. The need to improve IPC measures was highlighted by improving both healthcare staff’s IPC practices and the overall cleanliness of hospitals. Additionally, optimizing antibiotic use was emphasized, with special attention to issues like unindicated prescribing, inappropriate dosing and treatment duration, standardization of perioperative surgical prophylaxis, and promoting strategies such as de-escalation, regular prescription audits and feedback, and more frequent use of early-generation and topical antibiotics. A major recommendation was to develop department-specific antibiotic prescribing guidelines that are regularly updated to reflect local resistance patterns and include more cost-effective antibiotic options. Most participants stressed the importance of regular departmental meetings on AMR, training programs for doctors and medical staff, and collaboration with microbiology and pharmacology departments. Junior residents often pointed out that senior consultants should be regularly updated on new protocols and guidelines to endorse their implementation. Stricter regulation of antibiotic use was also seen as necessary, at the hospital level through stricter protocols, and at national level through tighter controls on antibiotic prescriptions in primary care and limits on OTC sales. Finally, the need for *“shift in the patients’ mindset and behavior” (O-05)* was emphasized, through public education and health campaigns focused on hygiene, antibiotics and AMR.

## Discussion

Our analysis identified several important factors perceived to influence antibiotic prescribing behavior among general and orthopedic surgeons in Central India. Environmental and sociocultural factors were found to play a vital role in the decision-making process around antibiotic prescribing. In addition, decisions regarding the type and duration of antibiotic treatment were multifactorial, strongly influenced by the prescriber´s experience and personal protocol. The need for feedback and consultation on antibiotic prescribing largely depended on the seniority level, with senior practitioners being more confident in their prescribing practices. Most participants emphasized the need to improve IPC measures and to regulate antibiotic prescribing by implementing department-specific guidelines tailored to local resistance patterns, as well as to standardize the practice of sending samples for culture and susceptibility testing. Many surgeons recalled the COVID-19 pandemic as a particularly chaotic period of inappropriate antibiotic use. AMR was perceived as a social problem that requires a collective effort.

In this study, the multifaceted nature of clinical reasoning around antibiotic prescribing is viewed through the lens of the COM-B-TDF framework, which provides a perspective on the complex interaction of cognitive capability, environmental resources, sociocultural context, and motivation. The WHO acknowledges the significant impact of sociocultural factors on health outcomes and recognizes that their systematic neglect is a major barrier to improving global health and living standards^34^. Previous research suggested that antibiotic prescribing behavior is influenced by social factors, such as perceived patients’ pressure and expectations, demand for a quick cure, lack of patients’ adherence, late presentation and loss to follow-up^6,9,11,35^. These factors were also emphasized by our study, alongside patients’ illiteracy and poor hygiene practices. However, in our study, the most critical factor affecting clinical decision-making on antibiotic prescribing was the socioeconomic status of patients, as the patients’ inability to afford certain antibiotics frequently forced the clinicians to provide suboptimal and possibly ineffective treatment. In addition, a dry and dusty rural environment was frequently emphasized as a factor contributing to frequent antibiotic prescribing as a precautionary measure.

The decision regarding the type and duration of antibiotic treatment was not only determined by clinical management knowledge and skills but also largely by the surgeon’s experience and personal preference. There were no prescribing guidelines, nor standardized and uniform protocol for antibiotic prescribing at the hospital levels. The choice of antibiotics was guided primarily by a positive experience with certain antibiotic regimens. These unwritten antibiotic prescribing protocols were passed on from senior consultants to the junior ones. Other studies have also suggested the presence of unwritten rules for clinical decisions^7,16^, which are influenced by personal, social, institutional and professional factors^36^ and can take precedence over guidelines adherence^16^. In our study, preferred antibiotic regimens consisted mostly of broad-spectrum antibiotics with inconsistent and non-standardized practices of de-escalation. However, it became evident during interviews that some participants were less familiar with the terms “broad- and narrow-spectrum antibiotics” and used the terms “lower/simple” and “higher” antibiotics instead, leading to the misclassification of certain antibiotics. Research shows that inappropriate antibiotic prescribing in surgery is common, with often extended duration of antibiotic treatment and surgical prophylaxis due to a conservative approach of infection risk reduction^7,15,17^. These were also seen as problematic in our study, with many surgeons questioning the need for prolonged surgical prophylaxis or antibiotic therapy but admitting that they did so due to fear of postoperative infections or patient loss to follow-up. However, increasing evidence does not support traditionally recommended long durations of antibiotic treatment for a number of indications, including intra-abdominal infections, urinary tract infections and cellulitis^30^.

In our study, junior surgeons expressed the need for more feedback, regulation and guidelines for antibiotic prescribing, whereas senior consultants were more confident in the empirical prescribing. However, confidence does not always translate into more appropriate prescribing practices^6^. Although most participants felt that their antibiotic prescribing was appropriate and mostly justified, previous research in the study setting showed suboptimal prescribing practices^21–23^. While it is expected that junior physicians may be less confident in their antibiotic prescribing due to a lack of clinical experience^6^, this study indicated the strong influence of professional hierarchy on decisions around antibiotic prescribing. Junior consultants emphasized that they did not feel empowered to suggest any changes in the current prescribing practices and that any intervention should first be endorsed by seniors to be implemented. Similar results were found in surgery departments in high-income settings^14,16^, which might suggest that professional hierarchy is a universally strong influencer on antibiotic prescribing in surgical practice. In general, the need for department-specific guidelines was repeatedly emphasized, especially by junior clinicians. The call for guidelines tailored to local resistance patterns was also expressed by other clinicians in India^6^ and junior surgeons in Australia^14^. Furthermore, prescription audits have not been conducted so far in the study setting, and the benefits of regular audits and feedback have been demonstrated in multiple studies^37–39^.

Culture and susceptibility testing was not consistently used to support antibiotic prescribing, and the timing, frequency and clinical reasons for sending samples differed among participants. While most surgeons agreed on the importance of culture and susceptibility testing, there seemed to be no standardized protocol nor routine practice around it. In particular, the recommended steps of reviewing initial empirical antibiotic therapy after one to three days, when more clinical and microbiological data become available, and considering the possibility of de-escalation^30,39^ were not systematically taken into account or implemented. Various reasons were given for not sending samples routinely for culture and susceptibility, such as long waiting time for the results, occasional lack of materials for sample collection, and mistrust in the results due to mishandling of samples. Previous research in India and other LMICs has shown that the main reasons for not sending samples for culture and susceptibility testing were a lack of laboratory facilities and time^6,35^, as well as the cost of laboratory tests^6^. However, these did not seem to be the main issues in our study, as participants were generally satisfied with the available laboratory services and subsidized cost, but the practice seemed to be more directed towards not seeing the need to send samples if empirical broad-spectrum antibiotics were effective. Additionally, the decision to send samples for culture and susceptibility and follow-up of cultures are often left to junior surgeons^17^, whereas in our study they expressed the need for more clarity and support in the process. Our analysis revealed a clear need for guidelines on clinical criteria, timing, and procedures for sample collection, regular staff training on their implementation, as well as a need from the laboratory side to expand the range of antibiotics tested for susceptibility.

The emergence of chaotic medical practices and antibiotic prescribing during COVID-19 as a topic highlights the profound impact the pandemic had on clinical practice and antibiotic misuse. India was highly affected by the pandemic and contributed a significant proportion to the global COVID-19 patient load^40^. The large misuse of medicines, in particular antibiotics, due to fear and the spread of misinformation about disease and treatment, has been well-documented globally^40,41^. Feelings of uncertainty and frustration over changing treatment protocols, challenges in keeping up with rapidly evolving evidence, combined with a heavy patient load and demanding clinical work, were very common among clinicians during the pandemic^40^. Antibiotics like azithromycin and doxycycline were largely prescribed with little or no evidence to relieve symptoms of COVID-19 and were frequently purchased OTC by the public for self-medication. As evidence evolved, the WHO advised against the routine use of azithromycin and doxycycline for COVID-19 treatment, except in cases of clear bacterial co-infection^40,41^. Antibiotic misuse during the pandemic has likely exacerbated already significant AMR burden in India, though the exact extent remains unclear^42^.

In general, surgeons were aware and concerned about AMR, although they were not familiar with the exact AMR burden in their setting. This view is supported by the results of a systematic review by Rezal et al.^6^; however, contrary to their findings, in our study, the AMR was not viewed as a primarily theoretical problem, as participants reported frequent challenges in treating resistant infections. Furthermore, surgeons were well aware of the profound impact of the AMR on every aspect of patients’ lives and expressed feelings of stress, frustration, hopelessness and failure when dealing with the AMR. Nevertheless, these negative emotions can also serve as a motivation for clinicians to improve antibiotic prescribing^43^. As raising awareness about AMR does not always translate into appropriate antibiotic prescribing, addressing environmental and sociocultural factors is equally important^44^.

This study suggests that AMR should be viewed more broadly as a social and environmental problem, rather than being limited to the medical field, which requires more political and public engagement. Antibiotics are known to be used as a “quick-fix” for unhygienic living conditions, lack of sanitation and IPC measures, as well as social inequalities^9,10,35^. Surgeons placed a main responsibility for AMR on others: primary and secondary healthcare providers, informal healthcare practitioners, unregulated pharmacy shopkeepers, and uninformed patients who “want miracles”. Blame-shifting among healthcare providers was reported by other studies as well^35^, with surgeons perceiving themselves to be less responsible than others for the AMR^14^. Similarly, in our study, surgeons saw themselves as the last-link of a complex chain of events that contribute to AMR, with the obligation to provide last-resort treatment to already resistant patients. Indeed, unregulated pharmacy sales and prescription of antibiotics by informal healthcare providers, as well as unindicated prescribing of antibiotics as a precautionary measure in primary care are well-documented factors contributing to the AMR in India^4,6,35^. However, the use of antibiotics as a precautionary measure is not limited to informal and primary-care providers; it is a common part of surgical practice also^12–17^. Therefore, many opportunities still exist to improve antibiotic prescribing in surgical practice^39^.

### Public health implications

This study proposed various strategies to address AMR, emphasizing the importance of a collective and multidisciplinary approach.

### Clinical practice recommendations

More attention to the IPC measures in hospitals should be given. Optimizing antibiotic prescribing practices was seen as necessary, including ensuring indication-based prescribing, appropriate dosing and duration of perioperative surgical prophylaxis and treatment regimens, routine de-escalation when suitable, and increased use of early-generation and topical antibiotics. The implementation of a standardized sample collection protocol, coupled with regular training and supervision of medical staff, was recommended. The development of contextualized department-specific antibiotic guidelines was highlighted as a priority. Guidelines are most useful if they are regularly updated based on local resistance patterns, involving multidisciplinary input from key stakeholders in their creation and implementation^6^, as well as including a greater selection of cost-effective antibiotic options. Regular prescription audits and feedback have proven to be beneficial for improving antibiotic use and reducing adverse events^6,39^. In general, hospitals should have an antibiotic stewardship program and a multidisciplinary antibiotic stewardship team^39^, with a dedicated staff member for antimicrobial stewardship within the surgical team^7^. Guidance from other specialists should not only be sought in complex cases, but a culture of routine collaboration with other departments, such as internal medicine, microbiology and pharmacology, should be fostered. Regular departmental meetings on AMR will be useful for information sharing, and continuous education of all medical staff, including senior surgeons, was proposed to improve awareness and practices.

### Policy recommendations

Stricter regulation of antibiotic use at both hospital and national levels was considered necessary. Public education campaigns on hygiene, antibiotics, and AMR were recommended to raise awareness and engage the broader community in addressing AMR.

### Methodological considerations

The strength of this study is that it is underpinned by the COM-B-TDF framework, which enables a holistic understanding of the antibiotic prescribing process by analyzing the clinical aspect of prescribing through cognitive, contextual, and social lenses, and ultimately providing a theory-informed interpretation of the data. The credibility and transferability of the study were strengthened by the detailed descriptions of the research setting and study participants. Triangulation of two independent researchers during data analysis and member checks increased the reliability and credibility of the study. It has to be noted that participants’ accounts were at times conflicting, as few participants changed their accounts throughout the course of the interviews.

The possibility of respondent bias cannot be excluded, as there was an element of social desirability in providing accounts about appropriate antibiotic prescribing practices. The interviews were conducted in English, so there was a possibility of a language barrier, as English was not the native language of either the interviewer or the participants. Further, although we aimed to conduct interviews in a quiet space and dedicated time, a few interviewees were interrupted and rushed during the interviews by clinical priorities. Nevertheless, all the interviews were finalized. In addition, few participants misclassified some of the broad-spectrum antibiotics (e.g. amoxiclav, cefotaxime, ceftriaxone) as narrow-spectrum or “lower” antibiotics, as well as misplaced some antibiotics into non-corresponding groups, which may have caused misunderstanding during certain parts of the interviews. Finally, our study included clinicians from three tertiary care hospitals in Central India; and while our findings may resonate with other similar settings, they do not capture the heterogeneity of India’s healthcare structure and may not be transferable to all settings.

## Conclusion

The findings of this study reveal the multifaceted nature of antibiotic prescribing among general and orthopedic surgeons in Central India. Antibiotic prescribing practices were predominately based on personal experience and profoundly impacted by environmental and sociocultural factors. Considering surgeons’ perspectives and the underlying factors influencing their antibiotic prescribing behavior is essential for designing context-specific targeted interventions, which should take seniority level into account. In addition, many opportunities to improve antibiotic use in orthopedic and surgical departments were highlighted, including strengthening IPC measures and diagnostic practices, developing and implementing contextualized antibiotic prescribing guidelines, and fostering regular interdepartmental communication and collaboration. AMR was seen as a complex social problem in which surgeons represent only a piece of the puzzle. Therefore, strategies to tackle AMR require a collective effort from the healthcare, political, and public sectors.

## Electronic supplementary material

Below is the link to the electronic supplementary material.

Supplementary Material 1

Supplementary Material 2

## Data Availability

The data that support the findings of this study are available from the corresponding author but restrictions apply to the availability of these data, which were used under license for the current study, and so are not publicly available. Data are however available from the corresponding author upon reasonable request and with permission of the Ethics Committee, R.D. Gardi Medical College, Ujjain, India.

## Abbreviations

AMR: antimicrobial resistance
AH: Avanti Hospital
CRGH: CR Gardi Hospital
IPC: Infection prevention and control
OTC: over-the-counter
SSIs: Surgical site infections
UCTH: Ujjain Charitable Trust Hospital
