# Supplementary material for: Antibiotic prescribing practices, perceived constraints, and views on antimicrobial resistance among general and orthopedic surgeons in central India
Source: Sci Rep. 2025 Jul 11;15:25099. doi: 10.1038/s41598-025-11173-w (PMC12254354; doi:10.1038/s41598-025-11173-w)
Supplement: Supplementary file 1 — Supplementary Material 1 [file 41598_2025_11173_MOESM1_ESM.docx]

**Supplementary material**

Table S1. Integrated Capability, Opportunity, Motivation-Behavior and the Theoretical Domains Framework (COM-B-TDF) model, developed by Cane et al., 2012.

| COM-B Component |  | TDF Domain |
| --- | --- | --- |
| Capability | Psychological | Skills |
|  |  | Knowledge |
|  |  | Memory, Attention and Decision Process |
|  |  | Behavioral Regulation |
|  | Physical | Skills |
| Opportunity | Social | Social Influences |
|  | Physical | Environmental Context and Resources |
| Motivation | Reflective | Social/Professional Role & Identity |
|  |  | Beliefs about Capabilities |
|  |  | Optimism |
|  |  | Beliefs about Consequences |
|  |  | Intentions |
|  |  | Goals |
|  | Automatic | Emotion |
|  |  | Reinforcement |

Table S2. Interview guide.

| **DECISION REGARDING ANTIBIOTIC PRESCRIBING** | |
| --- | --- |
| Please consider a scenario where a patient comes to your department with an injury which does not require operation. You are suspecting an infection, but you are not sure if the patient has an infection or not, or what organism might be causing it. | |
| 1. | How do you decide whether to prescribe an antibiotic? What influences your decision? |
| 2. | How do you make decision about which antibiotic substance to use in this case? |
| 3. | Which local or national guidelines for antibiotic prescribing do you follow? |
| 4. | In case of need, whom do you ask for advice on antibiotic prescribing? In which cases? |
| 5. | How do you know if you made a good decision about antibiotic prescribing? Do you get any feedback about your antibiotic prescribing practices? Would you like to get feedback, for example in the form of prescription audit reports? |
| **ANTIBIOTIC PRESCRIBING PRACTICES** | |
| 5. | Which antibiotics do you prescribe the most? Why? |
| 7. | What do you think about antibiotic prescribing practices in your department? |
| **CHOICE BETWEEN BROAD-SPECTRUM AND NARROW-SPECTRUM ANTIBIOTICS** | |
| 8. | How do you choose between narrow-spectrum and broad-spectrum antibiotics? |
| 9. | What are the benefits of prescribing broad-spectrum antibiotics versus narrow-spectrum antibiotics? What are the benefits of narrow-spectrum antibiotics? |
| 10. | How often do you switch to a narrow-spectrum antibiotic after prescribing broad-spectrum antibiotic? In which cases? Are you familiar with the term de-escalation? |
| 11. | What could be done to ensure appropriate prescribing of broad-spectrum antibiotics? |
| 12. | How would you comment on the use of broad-spectrum antibiotics in this department? What do you see as main barriers to appropriate use of broad-spectrum antibiotics in your department/hospital? (E.g. local culture, lack of laboratory facilities, organizational policies, external incentives or pressure, patient related factors- hygiene, infrastructure of settings, etc.) |
| **SENDING MICROBIOLOGY SAMPLES TO THE LABORATORY** | |
| 13. | How important do you think it is to send microbiology samples to the laboratory for culture and susceptibility testing? Why? |
| 14. | In which cases do you send microbiology samples to the laboratory? In which cases do you think there is no need to send microbiology samples to the laboratory? How often is it done in your department? How often should it be done in your department? |
| 15. | What do you see as the main barriers to more frequent use of laboratory facilities in this hospital? (E.g. long waiting time for results) |
| **ANTIMICROBIAL RESISTANCE** | |
| 16. | How often do you encounter cases of antimicrobial resistance in your practice? |
| 17. | How often do you encounter cases of superbugs in your department? Which ones do you encounter the most? (E.g. MRSA, Pseudomonas) |
| 18. | How does antimicrobial resistance influence your practice? |
| 19. | How often do you get information about the overall levels/burden of antimicrobial resistance in your department/hospital? |
| 20. | In your opinion, what could be done to reduce antimicrobial resistance in hospitals in general? |
| 21. | Did you work during the COVID-19 pandemic? What was your experience of antibiotic use and antimicrobial resistance during the COVID-19 pandemic? |
